# The Prognostic Role of Magnetic-Resonance-Imaging-Detected Corpus Invasion in Patients with Cervical Carcinoma Who Underwent Definitive or Adjuvant Pelvic Radiotherapy

**DOI:** 10.3390/cancers17091449

**Published:** 2025-04-26

**Authors:** Kuan-Ching Huang, Jen-Yu Cheng, Chung-Shih Chen, Chong-Jong Wang, Eng-Yen Huang

**Affiliations:** 1 Department of Radiation Oncology & Proton and Radiation Therapy Center, Kaohsiung Chang Gung Memorial Hospital, College of Medicine, Chang Gung University, Kaohsiung City 833, Taiwan; wilsonhkj@cgmh.org.tw (K.-C.H.); york480@cgmh.org.tw (J.-Y.C.); u780829@cgmh.org.tw (C.-S.C.); 2 Department of Radiation Oncology, Kaohsiung Municipal Ta-Tung Hospital, Kaohsiung City 801, Taiwan; 000y533@cgmh.org.tw; 3 School of Traditional Chinese Medicine, Chang Gung University, Taoyuan 333, Taiwan; 4 Department of Radiation Oncology, Kaohsiung Chang Gung Memorial Hospital, School of Medicine, College of Medicine, National Sun Yat-Sen University, Kaohsiung City 804, Taiwan

**Keywords:** uterine corpus invasion, cervical cancer prognosis, magnetic resonance imaging (MRI), pelvic radiotherapy, para-aortic lymph node recurrence, risk stratification

## Abstract

Objectives: In patients undergoing a radical hysterectomy, uterine corpus invasion worsens cervical cancer prognosis. However, the prognostic role of the invasion in locally advanced stages remains elusive. Due to the inadequacy of typical corpus biopsies, corpus invasion is diagnosed using magnetic resonance imaging (MRI). In this study, we investigated the prognostic role of MRI-detected uterine corpus invasion in patients undergoing radiotherapy for cervical cancer. Methods: This retrospective analysis involved 259 patients without extrapelvic metastases, diagnosed with FIGO 2009 stages IB–IVA cervical carcinoma from January 2011 to December 2020. The corpus invasion extent was classified as exocervical-confined (group 1), endocervical (group 2), or uterine corpus invasion (group 3). The rates of overall survival, cancer-specific survival, locoregional recurrence, para-aortic lymph node recurrence, and extrapelvic metastases after pelvic radiotherapy were analyzed. Kaplan–Meier and Cox regression analyses were used to determine recurrence-associated risks. Optimal risk stratification was predicted using a receiver operating characteristic curve with the area under the curve. Results: Groups 1, 2, and 3 included 66.0%, 18.9%, and 15.1% of patients, respectively. The 5-year para-aortic lymph node recurrence rates were 6.3%, 17.2%, and 34.2% (*p* < 0.001). Uterine corpus invasion was an independent factor for overall survival, cancer-specific survival, locoregional recurrence, extrapelvic metastases, and para-aortic lymph node recurrence. Including uterine corpus invasion in the risk stratification led to higher areas under the curve for overall survival, cancer-specific survival, locoregional recurrence, extrapelvic metastases, and para-aortic lymph node recurrence than using single parameters. Conclusions: In cervical cancer, following pelvic radiotherapy, uterine corpus invasion is a significant prognostic factor. More-aggressive treatments such as extended-field radiotherapy, adjuvant chemotherapy, and immune checkpoint inhibitors as an alternative to standard pelvic radiotherapy with concurrent chemotherapy may be considered in these patients.

## Simple Summary

Cervical cancer patients with uterine corpus invasion have a worse prognosis after surgery or radiation therapy. This study investigates how uterine corpus invasion affects recurrence patterns, showing that it is linked to a higher risk of para-aortic lymph node recurrence, locoregional relapse, and metastases beyond the pelvis. These findings suggest that incorporating corpus invasion into cervical cancer staging could help to identify patients who may benefit from more aggressive treatment, including extended-field radiotherapy. Understanding these risks can aid in optimizing treatment strategies to improve patient outcomes.

## 1. Introduction

Cervical cancer is the fourth most commonly diagnosed cancer and the fourth leading cause of cancer-related deaths among women, with an estimated 604,000 new cases and 342,000 deaths worldwide in 2020 [1]. Because of the advent of cervical smear screening and vaccine promotion, cervical cancer may be one of the most preventable cancers. However, uneven smear screening distribution and vaccination rates worldwide, along with socioeconomic disparities, contribute to higher risks in some regions [2]. Moreover, because of increasing late-stage disease and cervical adenocarcinoma incidences among young women, and persistent distant metastases, without a significant decline in mortality rates, cervical cancer is a globally prevalent cause of death [3]. Therefore, there is still a long way to go in cervical cancer treatment.

The 2018 FIGO [4,5] and 9th edition AJCC [6] staging systems are internationally recognized frameworks for clinical risk stratification in cervical cancer. Although previous studies have indicated that cervical cancer with uterine corpus invasion (UCI) is associated with significantly poorer overall survival (OS) [7,8,9,10], this prognostic factor is currently not incorporated into either staging system. Furthermore, patterns of treatment failure associated with UCI remain inconsistent, lacking a standardized approach to clearly identify or predict local recurrence and distant metastases. Increased para-aortic lymph node (PALN) recurrence is speculated as the cause [11,12], a hypothesis first proposed by Prempree [13] and Perez [14] based on dilatation and curettage during the initial workup to confirm cervical cancer UCI.

While postoperative pathological confirmation of corpus invasion in the early stage has been documented [15,16,17], such confirmation is lacking in advanced-stage patients undergoing concurrent chemoradiotherapy (CCRT) as the main treatment. Modern practices rarely involve dilatation and curettage, leading to the underdiagnosis of endometrial invasion [15]; therefore, magnetic resonance imaging (MRI) is the commonly available method for detecting corpus invasion at diagnosis.

This study aimed to evaluate the impact of uterine corpus invasion (UCI) on the prognosis following definitive or adjuvant radiotherapy, potentially refining staging and supporting prophylactic extended-field radiotherapy (EFRT).

## 2. Materials and Methods

### 2.1. Patient Characteristics

We retrospectively analyzed 259 patients with FIGO 2009 stage IB–IVA cervical carcinoma from January 2011 to December 2020. The inclusion criteria were definitive pelvic radiotherapy, radical hysterectomy with bilateral pelvic lymphadenectomy and adjuvant pelvic radiotherapy, and initial abdominopelvic MRI evaluation. Exclusion criteria were an Eastern Cooperative Oncology Group performance of 3–4 or an initial PALN metastasis diagnosed via laparotomy or image survey. Treatments followed institutional guidelines, including radical surgery for stage I–IIA and concurrent platinum-based CCRT for stage IIB–IVA cervical carcinoma. The characteristics of patients are shown in Table 1, in which the ‘Risk Group’ is defined based on a risk-scoring system [18,19] from our previous studies on EFRT indications. Pre-radiotherapy evaluations included physical examinations, SCC-Ag/CEA levels, and abdominopelvic MRIs. Positron emission tomography–computed tomography (PET/CT) scans were used for suspected MRI-positive lymph nodes or elevated tumor markers. Parametrial invasion was scored from 0 to 6 based on the tumor burden [20].

### 2.2. Measurement of Uterine Corpus Invasion

UCI was assessed using abdominopelvic MRIs with contrast and categorized into three groups based on tumor extension: group 1 (exocervical-confined, tumor localized at the ectocervix), group 2 (endocervical invasion, tumor extending up to the endocervical canal), and group 3 (uterine corpus invasion, tumor invading the uterine cavity from the squamocolumnar junction). The extent of tumor invasion was primarily evaluated using axial and sagittal views from T2-weighted and diffusion-weighted imaging (DWI) sequences. Image interpretation and diagnostic reports were completed by radiologists specialized in gynecologic oncology imaging as part of routine clinical practice. Furthermore, all gynecologic oncology patients underwent an interdisciplinary tumor board review, comprising radiologists, gynecologic oncologists, radiation oncologists, and medical oncologists, to comprehensively discuss and confirm disease staging and subsequent treatment strategies.

### 2.3. External Beam Radiotherapy

Treatment planning was based on CT scans, using conformal radiotherapy, intensity-modulated radiotherapy, or volumetric modulated arc therapy with Pinnacle or RayStation systems. Whole-pelvis fields were defined anatomically. The clinical target volume included the uterus, tumor, parametrium, pelvic sidewall, regional nodes, and vagina. Patients undergoing EFRT were excluded. Fractionation was five times per week at 1.8–2 Gy per fraction, with doses ranging from 39.6 to 45 Gy. For patients in whom brachytherapy applicator insertion was difficult to approach, additional boosts up to 66.6 Gy were delivered to the primary tumor. Patients with cervical carcinoma of stages IB2–IVA received concurrent cisplatin-based chemotherapy.

### 2.4. High-Dose-Rate Brachytherapy

Following external beam radiotherapy, 235 patients received high-dose-rate intracavitary brachytherapy using a microSelectron device with 192 Ir sources. A Henschke-type applicator was used with gauze packing mitigation for rectum and bladder exposure. Orthogonal imaging or pelvic CT scans were used for planning. Doses at Point A or the high-risk clinical target volume were 4.5–7 Gy (median: 6 Gy), administered biweekly in 2–6 fractions, totaling 12–36 Gy (median: 24 Gy).

### 2.5. Follow-Up

Patients were followed every three months for the first two years, then annually until death. Tumor markers were assessed at our routine follow-up. CT, MRI, or PET/CT scans were performed if tumor markers were elevated or when the patient presented with symptoms suggestive of recurrence. Overall survival (OS) referred to the time from diagnosis to death from any cause. Cancer-specific survival (CSS) referred to death due to cervical cancer. Recurrence is defined as the appearance of disease after achieving complete remission or progression of the residual tumor. Local recurrence (LR), regional recurrence (RR), and locoregional recurrence (LRR) referred to the times to primary tumor, pelvic lymph node, and tumor or lymph node recurrence, respectively. Para-aortic lymph node recurrence was diagnosed through CT, MRI, and PET/CT scans.

The PALN recurrence-free interval was determined by the following criteria [19]: (A) imaging confirming the lack of PALN recurrence evidence, (B) absence of symptoms like leg edema or sciatica, lack of recurrence, and normal SCC-Ag/CEA levels, and (C) survival for >10 years without regular follow-up. In the 2018 FIGO staging [4], a PALN metastasis is considered to be a locoregional disease; therefore, an extrapelvic metastasis (EPM) was defined as a PALN recurrence or other distant metastases. The time to PALN recurrence or an EPM was the interval from initial diagnosis to recurrence. We had developed a risk scoring system for extended-field radiotherapy in cervical SCC patients [19,20]. The high-risk group (score: 2) included those with SCC-Ag > 40 ng/mL, CEA > 10 ng/mL, or two to three minor factors (SCC-Ag 20–40 ng/mL, parametrial scores of 4–6, and positive pelvic nodes). Intermediate risk (score: 1) was defined by one minor factor, and low risk (score: 0) by the absence of risk factors. In the current study, incorporating the UCI degree (score: 0–2) into the previous system (score: 0–2) created a new risk scoring system (score: 0–4). The old and new scoring systems were compared for accuracy.

### 2.6. Statistical Analysis

Continuous variables (age, SCC-Ag, CEA, hemoglobin) were compared using an analysis of variance and Tukey’s post hoc test/the Games–Howell method. Proportional data were analyzed with chi-square or Fisher’s exact tests. Nonparametric Spearman’s correlation was tested for UCI and prognostic parameters. The Kaplan–Meier method and log-rank test were used for univariate analysis. A Cox regression model, avoiding collinearity, was used for multivariate analysis [21]. Hazard ratios (HRs) with 95% confidence intervals (CIs) quantified risk. The ROC curve identifies the best predictive models/clinical factors. Analyses were conducted using SPSS version 22.0. This study was reviewed by the Chang Gung Medical Foundation’s Institutional Review Board (202300965B0) who approved the waiver of participants’ consent due to the retrospective nature of the study.

## 3. Results

**Figure 1:**
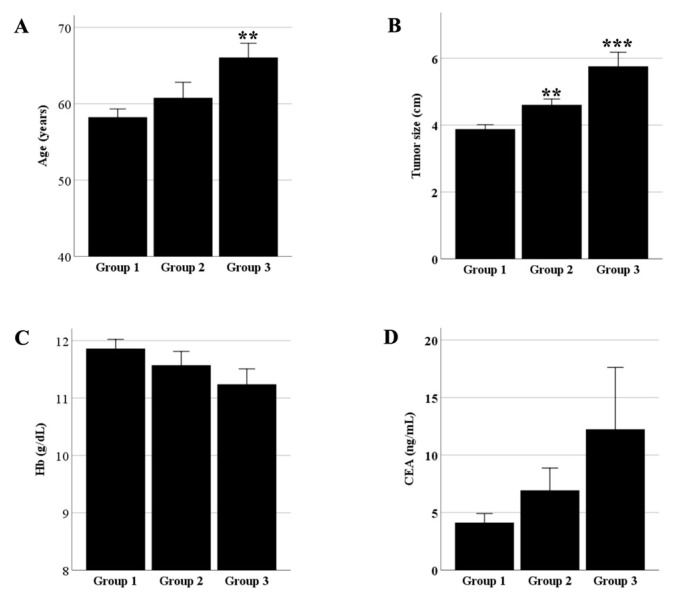
Analysis of variance for (**A**) age, (**B**) tumor size, (**C**) hemoglobin levels, and (**D**) carcinoembryonic antigen (CEA) levels in patients in groups 1, 2, and 3; ** and *** indicate *p* < 0.01 and <0.001, respectively, when compared with group 1.

Exocervical, endocervical, and corpus invasion were found in 171 (66.0%), 49 (18.9%), and 39 (15.1%) patients, respectively. Characteristics of the groups are shown in Table 1. The incidence of UCI was 7.2%, 17.7%, 18.5%, and 37.5% in patients with stage I, II, III, and IV cervical carcinoma. Characteristics of age ≥ 65 years (*p* = 0.002), tumor size ≥ 4 cm (*p* < 0.001), advanced stage (*p* < 0.001), parametrial score ≥ 4 (*p* = 0.001), hemoglobin < 12 g/dL (*p* = 0.002), and CEA ≥ 10 ng/mL (*p* = 0.005) correlated with UCI. Figure 1 shows the statistical significance for age (*p* = 0.004) and tumor size (*p* < 0.001), but not for hemoglobin (*p* = 0.172) and CEA levels (*p* = 0.307) between group 3 and group 1. Table 2 shows that UCI correlated with all prognostic parameters, with tumor size (0.332) having the highest correlation, followed by the parametrial score (0.248), and the lowest correlation was observed for SCC-Ag levels (0.152).

**Figure 2:**
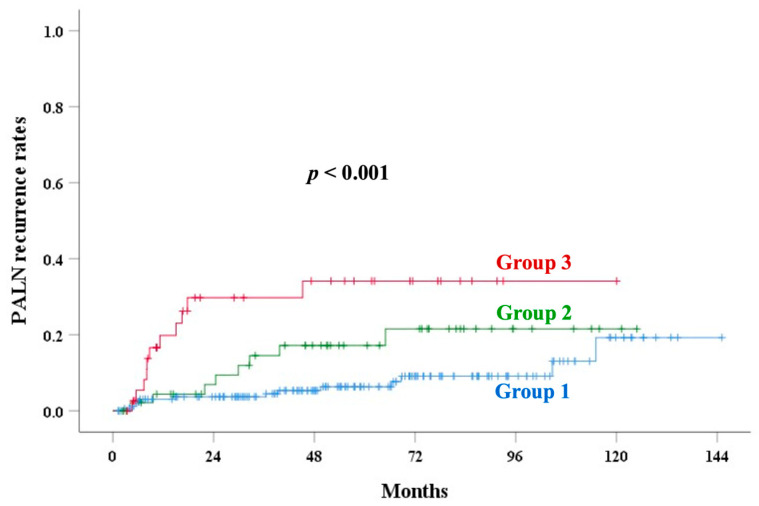
Para-aortic lymph node recurrence (PALNR) rates in various groups based on corpus invasion extent.

**Figure 3:**
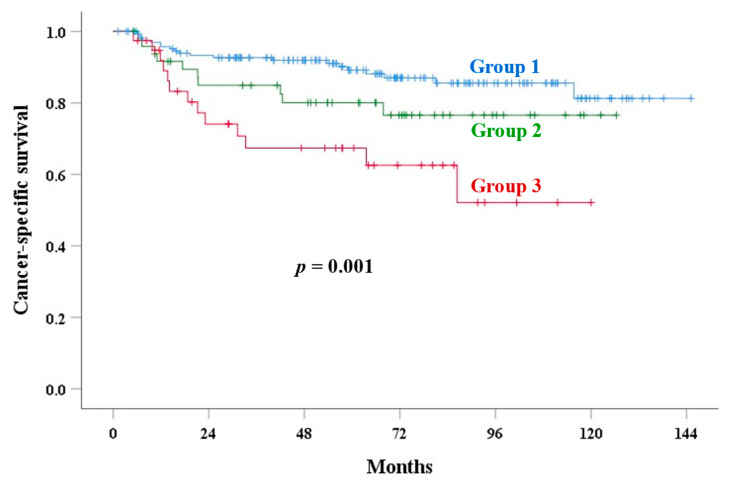
Cancer-specific survival in various groups based on corpus invasion extent.

**Figure 4:**
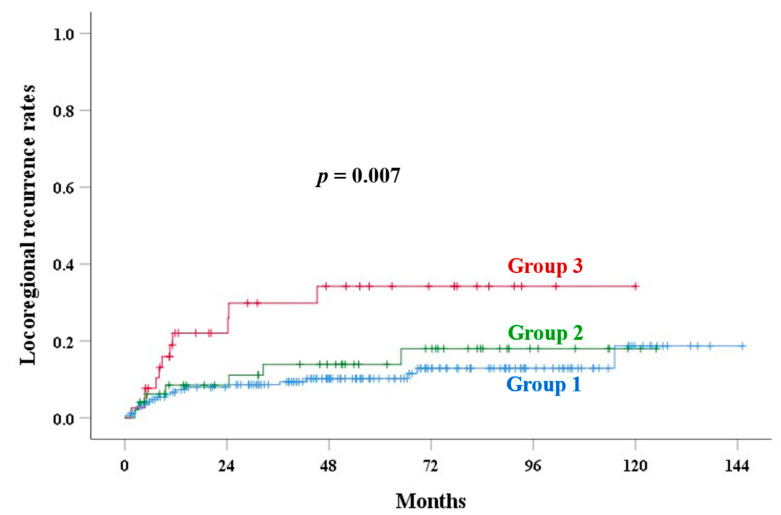
Locoregional recurrence rates in various groups based on corpus invasion extent.

**Figure 5:**
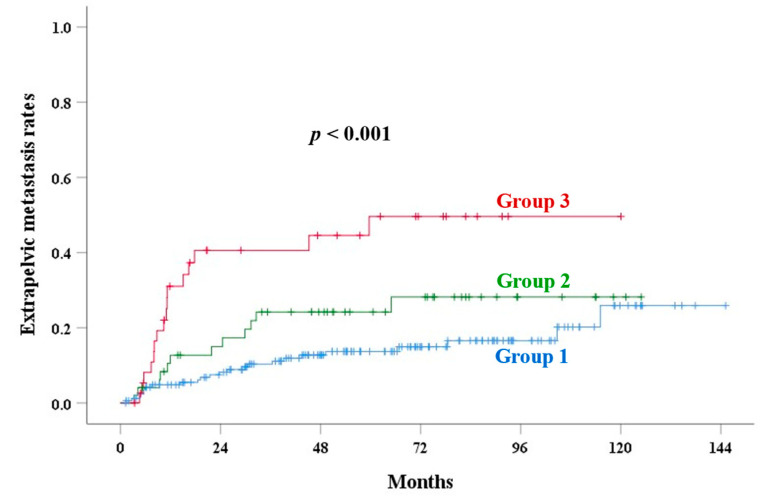
Extrapelvic metastasis rates in various groups based on corpus invasion extent.

The median follow-up time was 71.4 months (range: 4–145) in alive patients. Table 3 shows the univariate analysis of outcomes. The 5-year PALN recurrence rates of groups 1, 2, and 3 were 6.3%, 17.2%, and 34.2% (*p* < 0.001), respectively. UCI was a significant factor for OS (*p* < 0.001), PALNR (*p* < 0.001, Figure 2), CSS (*p* = 0.001, Figure 3), LRR (*p* = 0.007, Figure 4), and an EPM (*p* < 0.001, Figure 5). Table 4 and Table 5 show the multivariate analysis of outcomes. Corpus invasion remained a significant independent factor for OS (*p* < 0.001), CSS (*p* = 0.002), LRR (*p* = 0.010), an EPM (*p* < 0.001), and PALN recurrence (*p* = 0.001), with statistical trends for RR (*p* = 0.062) and LR (*p* = 0.078).

**Figure 6:**
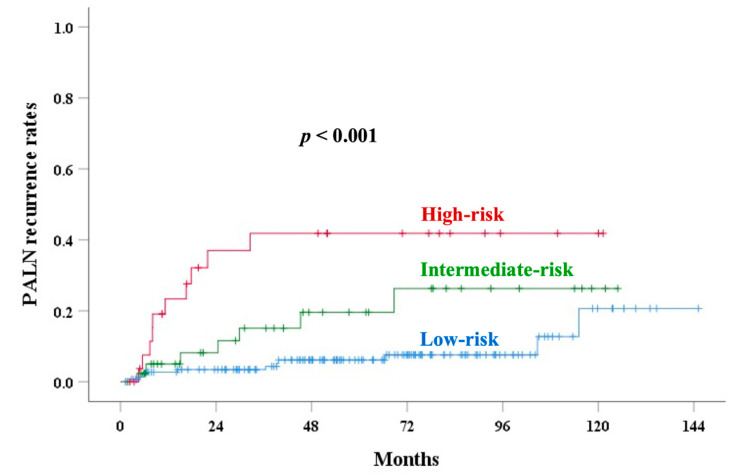
Para-aortic lymph node recurrence (PALNR) rates in various risk groups.

The ROC curve compared oncological outcomes of different prognostic parameters (Table 6 and Table 7). Except for RR, the new risk scoring had the leading AUC for CSS, LRR, an EPM, PALN recurrence, OS, and LLR. The 5-year PALN recurrence rates for new risk scores of 0 to 4 were 4.6%, 9.3%, 19.6%, 32.6%, and 55% (*p* < 0.001), respectively. HRs (95% CI) were 1.305 (0.381–4.466, *p* = 0.671), 2.913 (1.020–8.321, *p* = 0.046), 5.070 (1.607–16.001, *p* = 0.006), and 10.753 (3.387–34.142, *p* < 0.001) for scores 1, 2, 3, and 4, compared with score 0, respectively. Patients were classified into low- (scores: 0 and 1), intermediate- (score: 2), and high- (scores: 3 and 4) risk groups, with 5-year PALN recurrence rates of 6.1%, 19.6%, and 41.8% (*p* < 0.001, Figure 6), respectively.

## 4. Discussion

### 4.1. Summary of Main Results

The incidence of UCI increased with advancing stages. Several prognostic factors, such as old age, tumor size ≥ 4 cm, advanced stage, higher parametrial score, hemoglobin < 12 g/dL, and CEA ≥ 10 ng/mL, were significantly associated with UCI. Our results identified UCI as a significant independent factor for OS, CSS, LRR, an EPM, and PALN recurrence. By incorporating UCI into a new scoring system, stratifying patients into low-, intermediate-, and high-risk groups for PALN recurrence, the ROC curve demonstrated improved accuracy (Figure 2 and Figure 6).

### 4.2. Results in the Context of the Published Literature

Several studies have investigated the impact of UCI on cervical cancer [7,8,9,10,11,12,13,14,15,16], primarily focusing on surgically treated patients. However, confirming corpus invasion without endometrial curettage remains challenging in patients with locally advanced disease undergoing definitive CCRT. MRI has shown superiority over CT for assessing corpus invasion [22], with an overall diagnostic accuracy of approximately 95% [23], yet few studies utilized MRI specifically for prognostic evaluation [7,24].

Narayan et al. reported a high UCI rate (60%) among cervical cancer patients receiving definitive radiotherapy, which may partly explain their poorer outcomes (OS: 60%, 60%, 43%, and 14% for stages IB, II, III, and IVA) compared to our results (UCI rate: 13.5%; OS: 87.8%, 76.3%, 68.2%, and 25%, respectively) [7]. This discrepancy might reflect differences in radiotherapy techniques and disease stages. Our study further highlights correlations between UCI and PALN recurrence, emphasizing its prognostic importance. 

Wang et al. found prophylactic EFRT significantly improved 5-year OS, DFS, and PALN control in stage IIIC1 cases [25,26]. Similarly, a previous study also demonstrated improved outcomes following EFRT in patients at intermediate to high PALN recurrence risk [27]. Our findings indicate that patients who are clinically PALN-negative with UCI still exhibit a substantial PALN recurrence risk post-CCRT, potentially due to occult metastases.

Pathological evidence from surgical studies further supports the relationship between UCI and an initial PALN metastasis. Turan et al. analyzed patients with cancer of FIGO stages IB1–IIA undergoing radical surgery and found a significantly higher PALN metastasis rate in patients with UCI compared to those without (13.8% vs. 1.4%, *p* < 0.001) [16]. However, pathological PALN information is typically unavailable for patients with locally advanced disease, underscoring the importance of non-surgical evaluations such as PET/CT. Hope et al. noted a high PALN metastasis rate (30%) in patients with endometrial invasion detected by pathology and 18F-FDG PET, compared to 0% without invasion [11]. Another study by Huang et al. identified a prognostic role for MRI-detected corpus invasion, though without specific PALN recurrence data [24]. Their cohort predominantly included surgically treated patients with earlier-stage disease, potentially explaining their favorable outcomes relative to ours. 

Data from EMBRACE-I also confirmed an increased PALN recurrence risk in patients with uterine corpus involvement (10.7% vs. 6.1%, *p* = 0.004) [28]. Future trials, such as EMBRACE-III, may consider including MRI-detected UCI as an indication for elective PALN irradiation to enhance PALN control.

A plausible explanation for UCI promoting a distant metastasis is the myometrium’s extensive vascular and lymphatic network, facilitating cancer cell dissemination beyond the pelvis [24]. Matsuo et al. reported that UCI independently increased pelvic and possibly PALN metastasis risks, suggesting enhanced lymphatic spread [10]. Additionally, UCI likely reflects aggressive tumor biology, with non-squamous histologies potentially exhibiting distinct hematogenous metastatic patterns [10]. He et al. demonstrated that deep myometrial invasion (≥50%) significantly reduced disease-free survival and OS, likely due to increased cancer cell access to intramyometrial blood vessels and lymphatics, thereby facilitating lymphatic and hematogenous dissemination [15]. Similar mechanisms have been noted in endometrial carcinoma, where deeper invasion predicts lymphovascular space involvement and nodal micrometastases. This emphasizes the importance of invasion depth in terms of metastatic potential and recurrence risk [15]. Aggressive systemic therapy may be considered in patients with UCI. Although the OUTBACK trial [29] showed no survival benefit from adding adjuvant chemotherapy to CCRT, adjuvant systemic therapy may be considered for corpus invasion due to the 50% EPM rate. The KEYNOTE-A18 trial [30,31] demonstrated significant improvements in OS and PFS with the addition of pembrolizumab to CCRT. The hazard ratio was 0.89 for OS and 0.91 for PFS in IB2-IIB patients. In our patients with UCI, pembrolizumab may improve the outcomes because UCI was a poor prognostic factor for patients with FIGO stage IIB cervical carcinoma in our analysis.

Local recurrence occurs when cervical cancer spreads through embryonic structures in the uterovaginal compartment originating from the Müllerian anlage. These boundaries typically limit tumor expansion, but when breached, as in corpus invasion, it often indicates advanced disease [24,32,33]. Our results also revealed that most prognostic factors were significantly associated with corpus invasion (Table 2). Similar pathological prognostic factors, including older age, advanced stage, tumor size, adenocarcinoma, and parametrial invasion, were validated in 2212 cervical cancer patients who underwent a hysterectomy as being correlated with corpus invasion [34]. Our hypothesis on the correlation between increasing age and corpus invasion is based on squamocolumnar-junction positional changes. Higher estrogen levels make the junction more everted and visible on the ectocervix in younger, reproductive-age women [35]. However, with age and menopause, declining estrogen levels shift the junction into the endocervical canal, increasing the risk of invasion in the elderly. While the link between adenocarcinoma and invasion is not clear in biopsy results, our data showed that SCC/non-SCC distinctions did not significantly impact invasion because most biopsies only sampled SCC tissue from the ectocervix, leading to the potential underdiagnosis of endocervical adenocarcinoma/adenosquamous carcinoma. However, we found a significant correlation between corpus invasion and elevated CEA levels, as adenocarcinomas/adenosquamous carcinomas secrete more CEA than SCCs, explaining the lack of correlation between biopsy histology and invasion.

### 4.3. Strengths and Weaknesses

This study’s strength is its extensive use of MRI to predict treatment outcomes, particularly PALN recurrence. In patients with UCI, the LRR and risk of an EPM increased alongside PALN recurrence. For the early detection of residual tumor, endometrial curettage was considered three months after CCRT for patients in groups 2 and 3 who have MRI-detected deeply residual tumor behind the exocervix.

This study is limited by its retrospective design and some missing data. Additionally, MRI interpretations were performed by radiologists specializing in gynecologic oncology imaging as part of routine clinical practice, without blinded or independent double-read assessments, potentially introducing observer bias. While a previous study [19] emphasized SCC-Ag in PALN recurrence, we found no clear role for SCC-Ag because of the inclusion of non-SCC patients with low levels. However, patients with an SCC-Ag of ≥ 20 ng/mL received EFRT and were excluded from our study, reducing the inclusion of high SCC-Ag cases and weakening its predictive power. Initially, we considered subgroup analyses stratified by histology (SCC vs. non-SCC) and FIGO stage to evaluate potential variations in the prognostic significance of UCI. However, further subgrouping resulted in relatively small sample sizes, especially for advanced-stage cervical carcinoma (III–IV) and non-SCC histology. Given these limited subgroup numbers, performing additional subgroup analyses could substantially reduce the statistical power and potentially compromise the reliability of our findings.

### 4.4. Implications for Clinical Practice

Our findings underscore the prognostic significance of MRI-detected uterine corpus invasion (UCI) in cervical cancer patients undergoing radiotherapy. Specifically, patients with corpus invasion demonstrated a notably lower five-year overall survival rate (less than 60%), even compared to those with FIGO 2009 stage IIB cervical carcinoma. Recognizing the clinical relevance of corpus invasion can assist physicians in better identifying high-risk patients, guiding decisions on treatment intensification, personalized radiotherapy planning, and targeted follow-up strategies to improve patient outcomes.

### 4.5. Future Research

The results from our study suggest that future prospective, multicenter studies with larger patient cohorts are warranted to validate the prognostic importance of corpus invasion. Such research could explore whether formally incorporating corpus invasion as a sub-category (e.g., AJCC T2c or FIGO stage IIC) may further refine risk stratification and clinical management. Additionally, future investigations should clarify the biological mechanisms underlying the aggressive behavior associated with corpus invasion, and clinical trials assessing treatment strategies tailored for these high-risk patients would be valuable to enhance therapeutic effectiveness.

## 5. Conclusions

Uterine corpus invasion is a key prognostic factor of overall survival, cancer-specific survival, locoregional recurrence, para-aortic lymph node recurrence, and extrapelvic metastases after pelvic radiotherapy for cervical cancer. Extended-field radiotherapy, adjuvant chemotherapy, and immune checkpoint inhibitors may be considered for patients with uterine corpus invasion to improve treatment outcomes.

## Figures and Tables

**Table 1:** Patient characteristics.

	Group 1 (n = 171)	Group 2 (n = 49)	Group 3 (n = 39)	Total (n = 259)	*p*-Value
Parameter	n (%)	n (%)	n (%)	n (%)	
Age (y)					0.002
<65	114 (66.7)	30 (61.2)	15 (38.5)	159 (61.4)	
≥65	57 (33.3)	19 (38.8)	24 (61.5)	100 (38.6)	
Size (cm)					<0.001
<4	79 (46.2)	12 (24.5)	7 (17.9)	98 (37.8)	
≥4	64 (37.4)	34 (69.4)	30 (76.9)	128 (49.4)	
Unknown	28 (16.4)	3 (6.1)	2 (5.1)	33 (12.7)	
Stage					<0.001
I	68 (39.8)	9 (18.4)	6 (15.4)	83 (32.0)	
II	86 (50.3)	30 (61.2)	25 (64.1)	141 (54.4)	
III	14 (8.2)	8 (16.3)	5 (12.8)	27 (10.4)	
IV	3 (1.8)	2 (4.1)	3 (7.7)	8 (3.1)	
PM score					0.001
0–3	144 (84.2)	37 (75.5)	24 (61.5)	205 (79.2)	
4–6	13 (7.6)	8 (16.3)	8 (20.5)	29 (11.2)	
Unknown	14 (8.2)	4 (8.2)	7 (17.9)	25 (9.7)	
Pathology					0.534
SCC	144 (84.2)	43 (87.8)	34 (87.2)	221 (85.3)	
Non-SCC	27 (15.8)	6 (12.2)	5 (12.8)	38 (14.7)	
Hemoglobin (g/dL)					0.026
<12	67 (39.2)	25 (51.0)	23 (59.0)	115 (44.4)	
≥12	92 (53.8)	22 (44.9)	15 (38.5)	129 (49.8)	
Unknown	12 (7.0)	2 (4.1)	1 (2.6)	15 (5.8)	
Hypertension					0.046
No	119 (69.6)	30 (61.2)	21 (53.8)	170 (65.6)	
Yes	52 (30.4)	19 (38.8)	18 (46.2)	89 (34.4)	
Diabetes					0.667
No	142 (83.0)	44 (89.8)	30 (76.9)	216 (83.4)	
Yes	29 (17.0)	5 (10.2)	9 (23.1)	43 (16.6)	
Pelvic lymphadenopathy on MRI					0.062
Negative	142 (83.0)	36 (73.5)	28 (71.8)	206 (79.5)	
Positive	29 (17.0)	13 (26.5)	11 (28.2)	53 (20.5)	
Hysterectomy					0.761
No	162 (94.7)	47 (95.9)	38 (97.4)	247 (95.4)	
Yes	9 (5.3)	2 (4.1)	1 (2.6)	12 (4.6)	
Concurrent chemotherapy (cycles)					0.419
0	49 (28.7)	11 (23.4)	9 (23.1)	69 (26.6)	
1–3	7 (4.1)	1 (2.0)	3 (7.7)	11 (4.2)	
4–6	105 (61.4)	34 (69.4)	24 (61.5)	163 (62.9)	
>6	10 (5.8)	3 (6.1)	3 (7.7)	16 (6.2)	
SCC-Ag level (ng/mL)					0.285
<20	150 (87.7)	41 (85.1)	35 (89.7)	226 (87.3)	
20–40	12 (7.0)	3 (6.1)	0 (0.0)	15 (5.8)	
≥40	3 (1.8)	3 (6.1)	3 (7.7)	9 (3.5)	
Unknown	6 (3.5)	2 (4.1)	1 (2.6)	9 (3.5)	
CEA level (ng/mL)					
<10	157 (91.8)	41 (83.7)	31 (79.5)	229 (88.4)	0.005
≥10	8 (4.7)	6 (12.2)	7 (17.9)	21 (8.1)	
Unknown	6 (3.5)	2 (4.1)	1 (2.6)	9 (3.5)	
Risk group					0.110
Low	101 (59.1)	23 (46.9)	14 (35.9)	138 (53.3)	
Intermediate	28 (16.4)	10 (20.4)	9 (23.1)	47 (18.1)	
High	19 (13.5)	10 (20.4)	10 (25.6)	39 (15.1)	
Unknown	23 (13.5)	6 (12.2)	6 (15.4)	35 (13.5)	
EQD2 (Gy)					0.306
61–69.9	9 (5.6)	1 (2.1)	4 (10.5)	14 (5.7)	
70–79.9	19 (80.2)	36 (76.6)	26 (68.4)	192 (77.7)	
>80	23 (14.2)	10 (21.3)	8 (21.1)	41 (16.6)	

Abbreviations: SCC: squamous cell carcinoma, MRI: magnetic resonance imaging, SCC-Ag: squamous cell carcinoma antigen, EQD2: equivalent dose at 2 Gy.

**Table 2:** Correlation between corpus invasion extent and clinically prognostic parameters.

Parameter	Age	Stage	Size	PM Score	Hb	SCC	CEA
Age	1	-	-	-	-	-	-
Stage	0.130 (0.037)	1	-	-	-	-	-
Size	−0.088 (0.186)	0.502 (<0.001)	1	-	-	-	-
PM score	0.072 (0.274)	0.766 (<0.001)	0.558 (<0.001)	1	-	-	-
Hb	0.037 (0.564)	−0.305 (<0.001)	−0.368 (<0.001)	−0.273 (<0.001)	1	-	-
SCC	0.207 (0.001)	0.432 (<0.001)	0.418 (<0.001)	0.459 (<0.001)	−0.288 (<0.001)	1	-
CEA	0.235 (<0.001)	0.208 (0.001)	0.122 (0.068)	0.267 (<0.001)	−0.189 (0.004)	0.222 (0.001)	1
Corpus invasion	0.174 (0.005)	0.245 (<0.001)	0.332 (<0.001)	0.248 (<0.001)	−0.165 (0.010)	0.152 (0.016)	0.214 (0.001)

**Table 3:** Univariate overall survival (OS), cancer-specific survival (CSS), locoregional recurrence (LRR), extrapelvic metastasis (EPM), and para-aortic lymph node recurrence (PALNR) analyses.

Parameter	OS	*p*-Value	CSS	*p-*Value	LRR	*p*-Value	EPM	*p*-Value	PALNR	*p*-Value
Age (y)		0.006		0.707		0.459		0.526		0.072
<65	81.9		83.4		14.7		21.2		14.9	
≥65	70.7		86.7		13.7		21.6		7.9	
Size (cm)		0.030		0.004		0.132		0.016		0.011
<4	83.3		92.4		11.8		17.4		8.8	
≥4	70.4		74.5		18.0		28.3		18.0	
Stage		<0.001		<0.001		0.001		0.016		0.002
I	87.8		95.9		7.2		9.7		7.1	
II	76.3		82.7		14.0		24.3		14.6	
III	68.2		73.1		25.7		32.1		8.6	
IV	25.0		28.6		51.1		55.6		55.6	
PM score [20]		0.030		0.001		0.019		0.006		0.002
0–3	81.8		89.7		12.3		16.6		9.3	
4–6	58.2		61.7		28.8		44.3		32.6	
Pathology		0.605		0.122		0.024		0.068		0.458
SCC	77.6		85.3		12.8		19.6		12.4	
Non-SCC	77.2		79.8		22.0		29.3		13.1	
Hemoglobin (g/dL)		<0.001		0.054		0.019		0.005		0.124
<12	68.1		78.1		20.1		31.2		16.6	
≥12	87.5		88.3		9.3		14.6		10.1	
Pelvic lymphadenopathy		0.028		0.002		0.003		0.178		0.011
Negative	80.6		88.3		11.1		19.5		9.8	
Positive	65.7		69.9		26.3		27.3		23.1	
Corpus invasion		<0.001		0.001		0.007		<0.001		<0.001
Group 1	82.0		89.2		10.2		13.7		6.3	
Group 2	77.6		80.1		13.9		24.2		17.2	
Group 3	57.3		67.4		34.2		49.6		34.2	
SCC-Ag level (ng/mL)		0.360		0.001		0.051		0.607		0.132
<20	79.1		85.8		12.4		20.3		11.5	
20–40	71.4		78.6		21.2		21.4		9.1	
≥40	66.7		75.0		37.5		37.5		37.5	
CEA level (ng/mL)		0.324		0.338		0.009		0.075		0.081
<10	77.4		84.3		13.3		20.3		12.0	
≥10	67.4		77.1		28.6		31.9		25.0	

Scoring for parametrial (PM) involvement [20] was based on the concept of tumor burden. In brief, scores of 0, 1, 2, or 3 were given to each side with no PM, medial PM, lateral PM, or sidewall involvement, respectively. The total score ranged from 0 to 6.

**Table 4:** Multivariate cancer-specific survival (CSS), locoregional recurrence (LRR), extrapelvic metastasis (EPM), and para-aortic lymph node recurrence (PALNR) analyses.

Parameter	CSS HR (95% CI)	*p*-Value	LRR HR (95% CI)	*p*-Value	EPM HR (95% CI)	*p*-Value	PALNR HR (95% CI)	*p*-Value
Pathology		0.218		0.049		0.088		0.524
Non-SCC	Reference		Reference		Reference		Reference	
SCC	0.607 (0.274–1.344)		0.435 (0.190–0.997)		0.534 (0.260–1.098)		0.727 (0.272–1.941)	
Extent of corpus invasion		0.002		0.010		<0.001		0.001
Exocervical	Reference		Reference		Reference		Reference	
Endocervical	1.564 (0.715–3.422)	0.263	1.163 (0.478–2.830)	0.739	1.729 (0.853–3.506)	0.129	1.858 (0.749–4.607)	0.181
Uterine corpus	3.743 (1.820–7.700)	<0.001	3.259 (1.491–7.124)	0.003	4.369 (2.271–8.406)	<0.001	5.089 (2.211–11.709)	<0.001
Pelvic lymph node		0.019		0.037		0.517		0.080
Negative	Reference		Reference		Reference		Reference	
Positive	2.205 (1.138–4.275)		2.163 (1.049–4.460)		1.239 (0.648–2.371)		2.005 (0.919–4.732)	
SCC-Ag level		0.257		0.071		0.672		0.391
0–20 ng/mL	Reference		Reference		Reference		Reference	
20–40 ng/mL	2.606 (0.873–7.778)	0.086	3.582 (1.168–10.983)	0.026	1.736 (0.517–5.831)	0.372	1.916 (0.424–8.653)	0.398
≥40 ng/mL	1.385 (0.318–6.025)	0.664	2.263 (0.637–8.031)	0.206	1.696 (0.502–5.732)	0.395	2.391 (0.677–8.446)	0.176
Unknown	2.004 (0.537–7.474)	0.301	2.094 (0.555–7.903)	0.276	1.308 (0.359–4.770)	0.684	1.960 (0.407–9.449)	0.402

**Table 5:** Multivariate overall survival (OS), regional recurrence (RR), and LR analyses.

Parameter	OS HR (95% CI)	*p*-Value	RR HR (95% CI)	*p*-Value	LR HR (95% CI)	*p*-Value
Pathology		0.856		0.079		0.157
Non-SCC	Reference		Reference		Reference	
SCC	0.935 (0.451–1.937)		0.285 (0.070–1.159)		0.495 (0.187–1.312)	
Extent of corpus invasion		<0.001		0.062		0.078
Exocervical	Reference		Reference		Reference	
Endocervical	1.165 (0.591–2.299)	0.659	1.563 (0.353–6.930)	0.557	0.931 (0.302–2.873)	0.901
Uterine corpus	3.321 (1.858–5.937)	<0.001	4.829 (1.279–18.234)	0.020	2.775 (1.096–7.028)	0.031
Pelvic lymph node		0.073		0.058		0.069
Negative	Reference		Reference		Reference	
Positive	1.684 (0.953–2.974)		3.332 (0.959–11.577)		2.208 (0.941–5.180)	
SCC-Ag level		0.246		0.048		0.727
0–20 ng/mL	Reference		Reference		Reference	
20–40 ng/mL	2.084 (0.804–5.399)	0.131	3.209 (0.340–30.331)	0.309	3.426 (0.949–12.366)	0.060
≥40 ng/mL	1.486 (0.448–4.929)	0.517	8.153 (1.724–38.548)	0.008	<0.001 (<0.001–)	0.977
Unknown	2.097 (0.675–6.511)	0.200	2.473 (0.249–24.561)	0.440	1.898 (0.376–9.566)	0.438

**Table 6:** Cancer-specific survival (CSS), locoregional recurrence (LRR), extrapelvic metastasis (EPM), and para-aortic lymph node recurrence (PALNR) receiver operator characteristic (ROC) curves.

Parameter	CSS AUC (95% CI)	*p*-Value	LRR AUC (95% CI)	*p*-Value	EPM AUC (95% CI)	*p*-Value	PALNR AUC (95% CI)	*p*-Value
Age	0.427 (0.333–0.522)	0.131	0.417 (0.321–0.514)	0.108	0.434 (0.349–0.519)	0.139	0.399 (0.299–0.499)	0.065
Pathology	0.449 (0.350–0.547)	0.287	0.428 (0.322–0.534)	0.161	0.447 (0.356–0.538)	0.241	0.477 (0.367–0.586)	0.670
SCC	0.621 (0.527–0.714)	0.016	0.592 (0.491–0.693)	0.084	0.592 (0.505–0.678)	0.047	0.594 (0.486–0.702)	0.095
CEA	0.581 (0.483–0.679)	0.095	0.637 (0.540–0.735)	0.008	0.605 (0.515–0.695)	0.021	0.607 (0.502–0.713)	0.050
Hb	0.583 (0.488–0.679)	0.087	0.632 (0.534–0.729)	0.013	0.617 (0.530–0.704)	0.010	0.584 (0.476–0.692)	0.125
Size	0.642 (0.547–0.738)	0.005	0.565 (0.458–0.672)	0.230	0.625 (0.539–0.711)	0.008	0.634 (0.536–0.731)	0.020
LN	0.600 (0.502–0.699)	0.038	0.601 (0.496–0.706)	0.048	0.540 (0.450–0.630)	0.368	0.597 (0.485–0.709)	0.075
Stage	0.654 (0.562–0.746)	0.001	0.610 (0.507–0.712)	0.033	0.595 (0.511–0.679)	0.035	0.547 (0.441–0.654)	0.385
PM score	0.654 (0.552–0.756)	0.003	0.590 (0.478–0.701)	0.095	0.639 (0.551–0.727)	0.003	0.615 (0.499–0.730)	0.046
Extent of corpus invasion	0.630 (0.534–0.727)	0.007	0.602 (0.497–0.707)	0.048	0.639 (0.550–0.728)	0.002	0.661 (0.553–0.768)	0.003
Old risk scoring	0.658 (0.556–0.7861)	0.003	0.698 (0.595–0.801)	<0.001	0.593 (0.497–0.690)	0.053	0.655 (0.544–0.765)	0.008
New risk scoring	0.713 (0.626–0.800)	<0.001	0.714 (0.620–0.807)	<0.001	0.640 (0.547–0.733)	0.004	0.692 (0.577–0.807)	0.001

**Table 7:** Overall survival (OS), regional recurrence (RR), and LR receiver operator characteristic (ROC) curves.

Parameter	OS AUC (95% CI)	*p*-Value	RR AUC (95% CI)	*p*-Value	LR AUC (95% CI)	*p*-Value
Age	0.600 (0.513–0.688)	0.016	0.449 (0.288–0.609)	0.532	0.381 (0.270–0.492)	0.047
Pathology	0.483 (0.401–0.566)	0.688	0.415 (0.243–0.587)	0.303	0.432 (0.308–0.555)	0.255
SCC	0.636 (0.559–0.712)	0.002	0.698 (0.543–0.853)	0.020	0.503 (0.388–0.618)	0.963
CEA	0.603 (0.522–0.685)	0.014	0.631 (0.478–0.784)	0.112	0.614 (0.492–0.735)	0.058
Hb	0.650 (0.570–0.730)	<0.001	0.746 (0.625–0.867)	0.003	0.585 (0.463–0.706)	0.173
Size	0.565 (0.475–0.654)	0.143	0.553 (0.357–0.749)	0.573	0.597 (0.478–0.716)	0.113
LN	0.561 (0.478–0.645)	0.142	0.676 (0.510–0.841)	0.033	0.579 (0.456–0.701)	0.188
Stage	0.616 (0.536–0.696)	0.005	0.582 (0.415–0.749)	0.318	0.613 (0.497–0.730)	0.058
Pm score	0.599 (0.512–0.686)	0.024	0.557 (0.364–0.750)	0.506	0.582 (0.454–0.710)	0.198
Extent of corpus invasion	0.615 (0.531–0.699)	0.006	0.665 (0.502–0.828)	0.045	0.562 (0.438–0.685)	0.302
Old risk group	0.588 (0.499–0.677)	0.049	0.724 (0.556–0.892)	0.012	0.667 (0.548–0.786)	0.010
New risk group	0.662 (0.582–0.742)	<0.001	0.735 (0.571–0.899)	0.009	0.675 (0.571–0.779)	0.007

## Data Availability

In accordance with the journal’s guidelines, data supporting the findings of this study will be made available upon reasonable request. The data can be accessed for independent analysis by a selected team designated by the Editorial Team for further validation, additional data analysis, or reproducibility in other centers, if required.

## Abbreviations

The following abbreviations are used in this manuscript:

Abbreviation	Full Name
MRI	Magnetic Resonance Imaging
FIGO	International Federation of Gynecology and Obstetrics
AJCC	American Joint Committee on Cancer
PALN	Para-aortic Lymph Node
CCRT	Concurrent Chemoradiotherapy
UCI	Uterine Corpus Invasion
EFRT	Extended-Field Radiotherapy
SCC-Ag	Squamous Cell Carcinoma Antigen
CEA	Carcinoembryonic Antigen
PET/CT	Positron Emission Tomography/Computed Tomography
OS	Overall Survival
CSS	Cancer-Specific Survival
LR	Local recurrence
RR	Regional recurrence
LRR	Locoregional Recurrence
EPM	Extrapelvic Metastasis
PALNR	Para-aortic Lymph Node Recurrence
CT	Computed Tomography
HR	Hazard Ratio
CI	Confidence Interval
ROC Curve	Receiver Operating Characteristic Curve
AUC	Area Under the Curve
EQD2	Equivalent Dose at 2 Gy
